# Supplementary material for: Intrinsic neuronal properties represent song and error in zebra finch vocal learning
Source: Nat Commun. 2020 Feb 19;11:952. doi: 10.1038/s41467-020-14738-7 (PMC7031510; doi:10.1038/s41467-020-14738-7)
Supplement: Supplementary file 1 — Supplementary Information [file 41467_2020_14738_MOESM1_ESM.pdf]

Supplementary Information:

Intrinsic neuronal properties represent song and error in zebra finch vocal learning

Arij Daou[1†], Daniel Margoliash[1,2]

[1]Department of Organismal Biology & Anatomy, University of Chicago, IL 60637 USA.

[2]Grossman Institute for Neuroscience, Quantitative Biology and Human Behavior, University of Chicago

[†]Current address: Biomedical Engineering Program, American University of Beirut, Beirut, Lebanon.

Materials and corresponding author: D.M. (dan@bigbird.uchicago.edu)

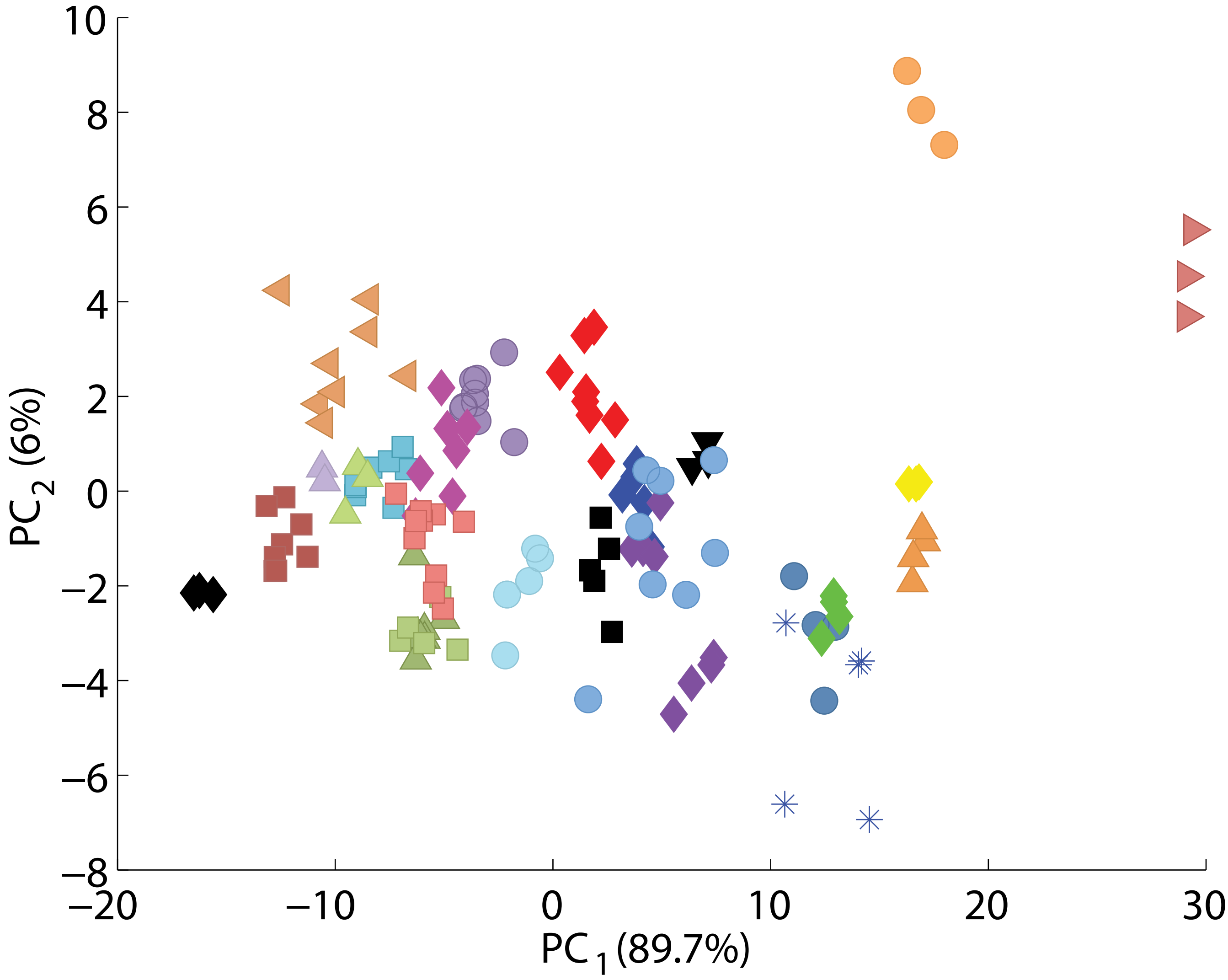

Supplementary Figure 1. Principal component analysis emphasizes across-bird differences. PCA of the first three ISIs and spike rate separates into separate clusters the 33 birds with two or more cells remaining after eliminating all cells with < 4 spikes (each bird a different color/symbol). PC1 and PC2 accounted for 89.7% and 6%, respectively, of the variance across birds. After normalizing the data to [-1, 1], the loadings are spike rate (-4.27) and ISI 1-3 (3.37, 6.08, 11.99).

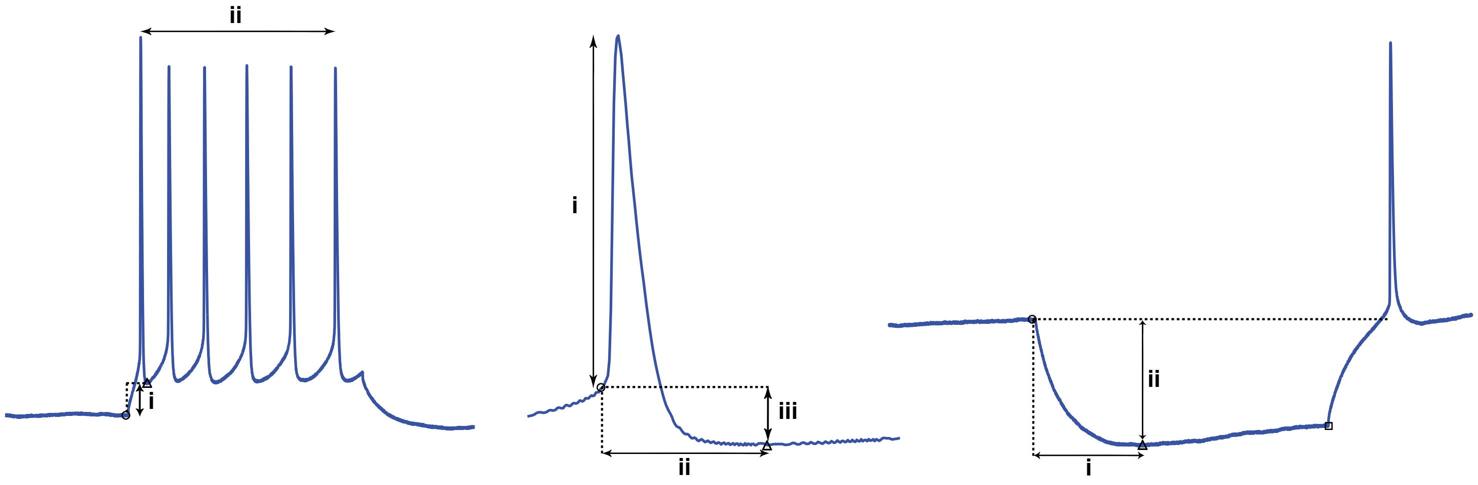

Supplementary Figure 2. Features extracted from waveforms illustrating how the parameters were measured. Left panel Voltage trace from a X-projecting neuron (bird White11) responding to a 100 pA depolarizing current pulse. **i.** Plateau amplitude measured from the voltage at the onset of applied current (circle) to the nadir of the AHP of the first spike (triangle) **ii.** Spike rate measured as the duration from the peak of the first spike to the peak of the last spike divided by the number of spikes. **Middle panel** First spike of the same neuron in the left panel depicting the first spike extracted features. **i.** Spike amplitude **ii.** Time to peak (TTP) hyperpolarization **iii.** Spike after hyperpolarization (AHP). Circle represents the spike threshold, calculated as the peak of the instantaneous second derivative of the spike voltage trace. The triangle represents the voltage at the nadir of the spike. **Right Panel** Voltage response of the same neuron in the left panel to a -120 pA hyperpolarizing current pulse illustrating the features extracted from the sag. **i.** Time to peak (TTP) sag **ii.** Amplitude of peak hyperpolarization (APH). Circle represents the voltage at the onset of the applied current, triangle represents the voltage at the nadir (V_{min}), and square represents the voltage at the end of the current pulse (V_{end}). Note the rebound spike.

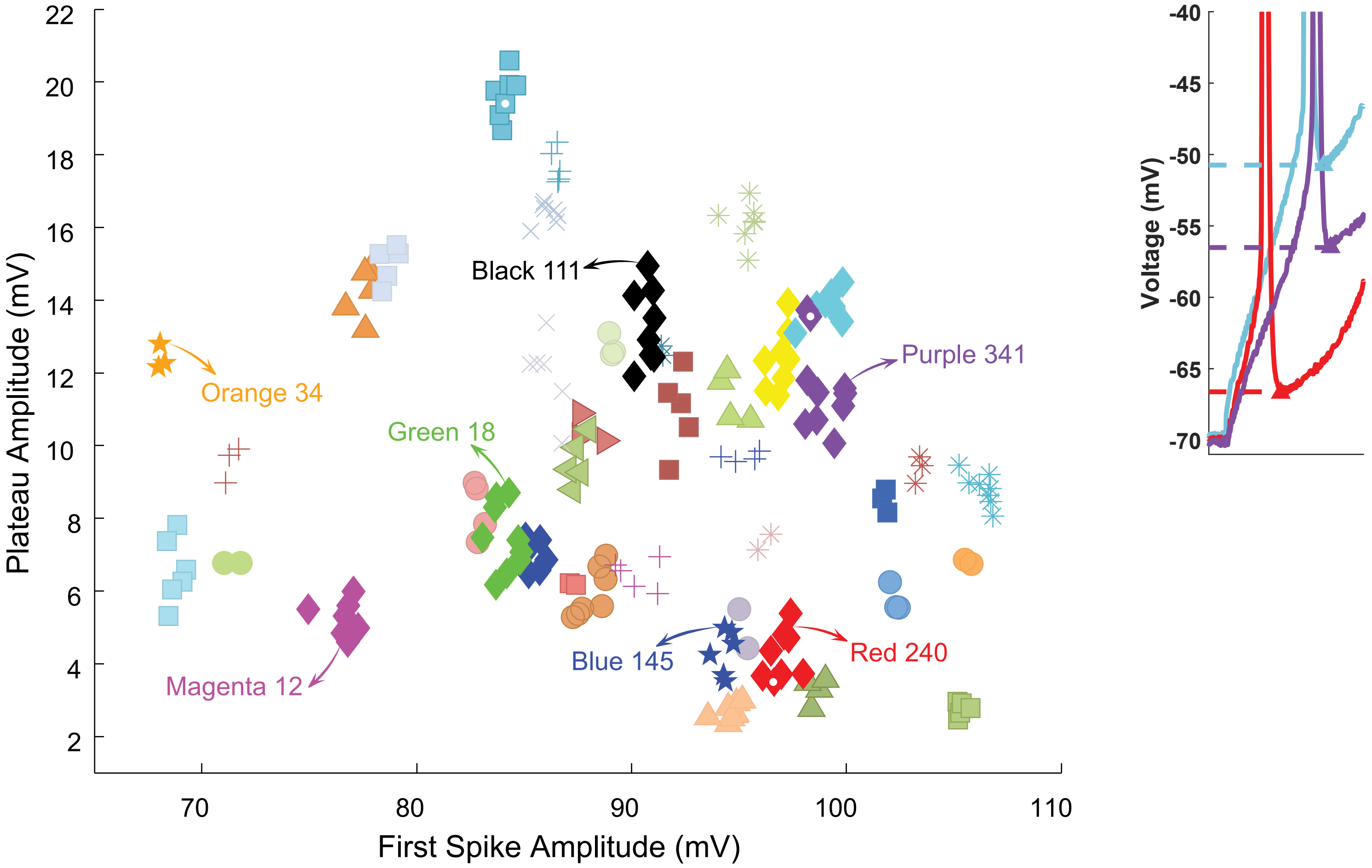

Supplementary Figure 3. Additional electrophysiological measures showing clustering across birds. Scatter plot for first spike amplitude versus plateau amplitude illustrates homogeneity within neurons of the same birds and a clear segregation across neurons of different birds. The visual impression is statistically significant (see Methods, Additional statistical analysis, Supplemental Fig. 3). The inset shows measurement of plateau amplitude for three neurons, with each neuron identified with a white dot in the plot. Similar segregation was seen for other features such as AHP and sag ratio (data not shown), but the distinction wasn't as pronounced as first spike amplitude, threshold and plateau amplitude. Symbols and colors represent the same neurons and birds as in Fig. 1b.

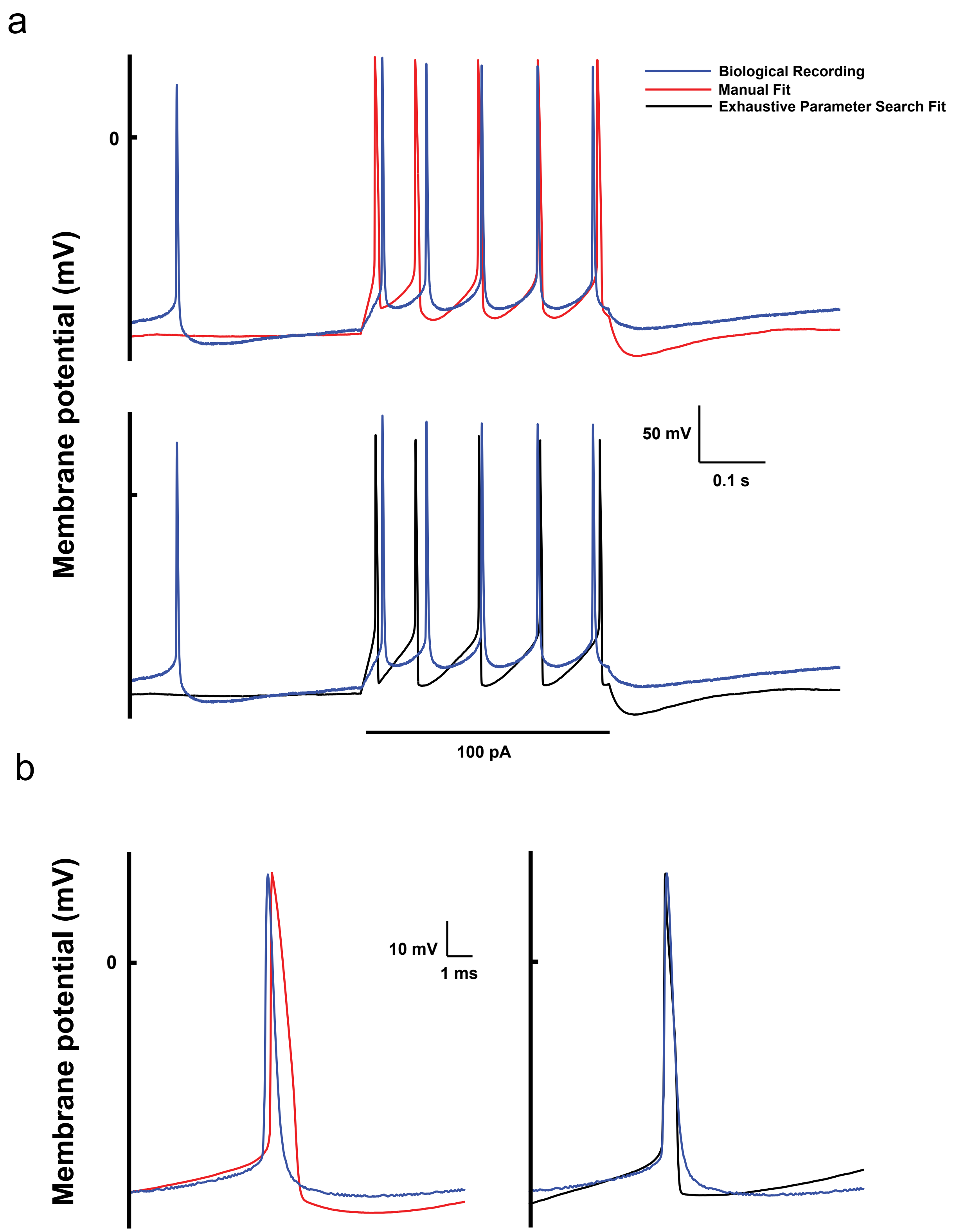

Supplementary Figure 4. Comparison of manual and automated fits illustrating the biological discrepancies of the brute force search results. a Top panel shows overlaid voltage traces of the biological recording (blue) and manual fit simulation (red). Bottom panel shows overlaid waveforms for the same biological recording (blue) and the best fit for the automated parameter search (black). Both manual and automated fits generated good estimates for spike amplitude, spike frequency, spike timing, and overall spike morphology. The manual fit, however, elicited a more realistic looking trace when compared to the biological recording in terms of its plateau amplitude and the smooth AHP following action potentials. **b** Overlaid first spike waveforms for both manual (left, red) and automated (right, black) fits with the biological first spike waveform (blue). The automated fit's first spike matched better the spike duration and spike amplitude features; the manual fit matched better the spike AHP and plateau amplitude. The APH shape of the automated fit is aberrant (see Methods: Exhaustive parameter searches).

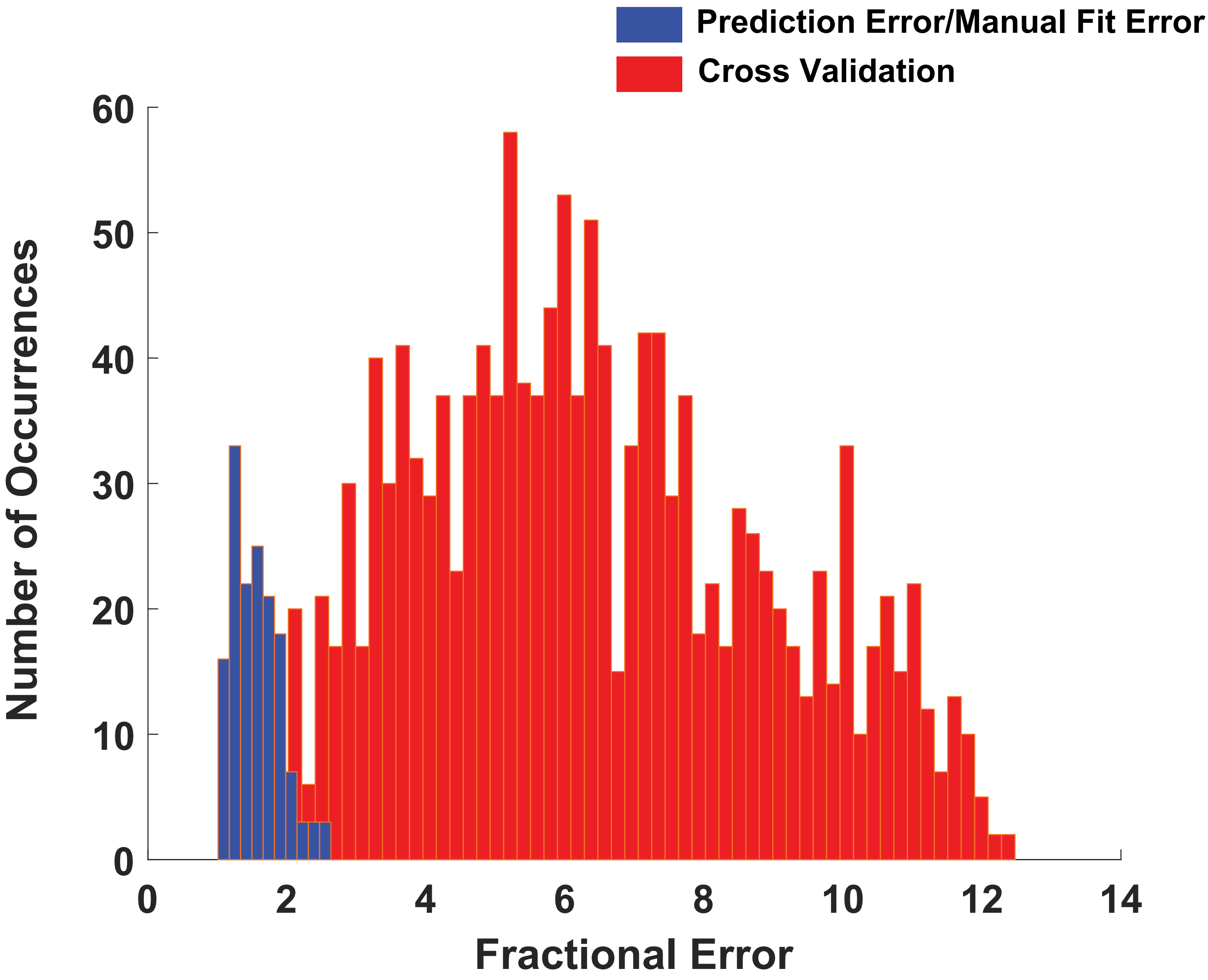

Supplementary Figure 5. Model predictions and cross validation. Cross-validation analyses demonstrates that the model of a given neuron was far better at predicting responses of that neuron than were models of neurons from other birds. The error function used mean squares to compute the difference between the biological voltage trace and the model simulation by adding the individual differences between the two for each of the following features: plateau amplitude, spike amplitude, spike duration, number of spikes, the timing of each individual spike, resting membrane potential, sag ratio and rebound firing.

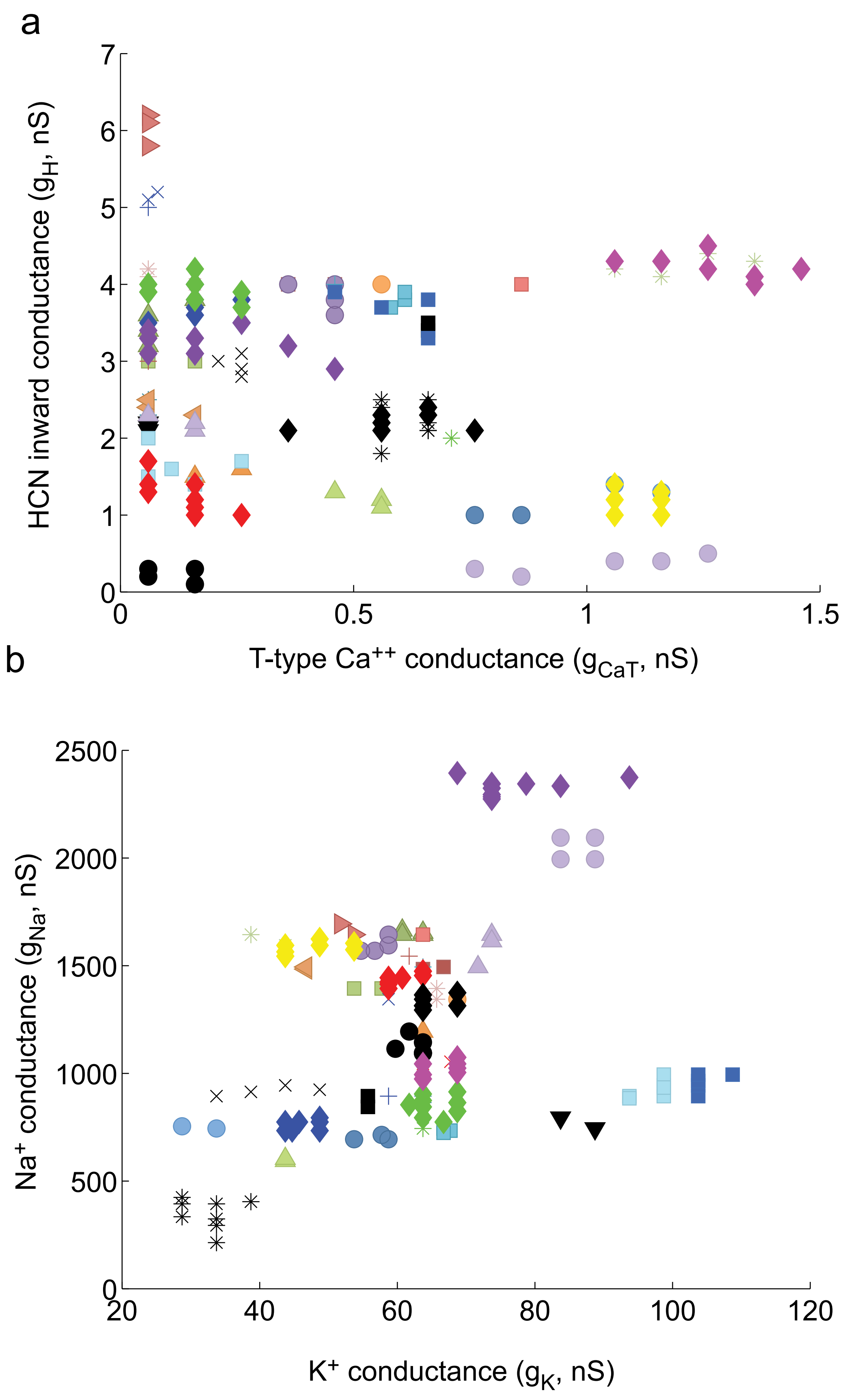

Supplementary Figure 6. Manual fitted data shows clustering for other parameters. Scatter plots for conductance maximum values derived from manual fits, **a** $g_{Ca\text{-}T}$ vs. g_H, and **b** g_K vs. g_{Na}, shows clusters of values for neurons of each bird and wide variation of cluster positions for different birds.

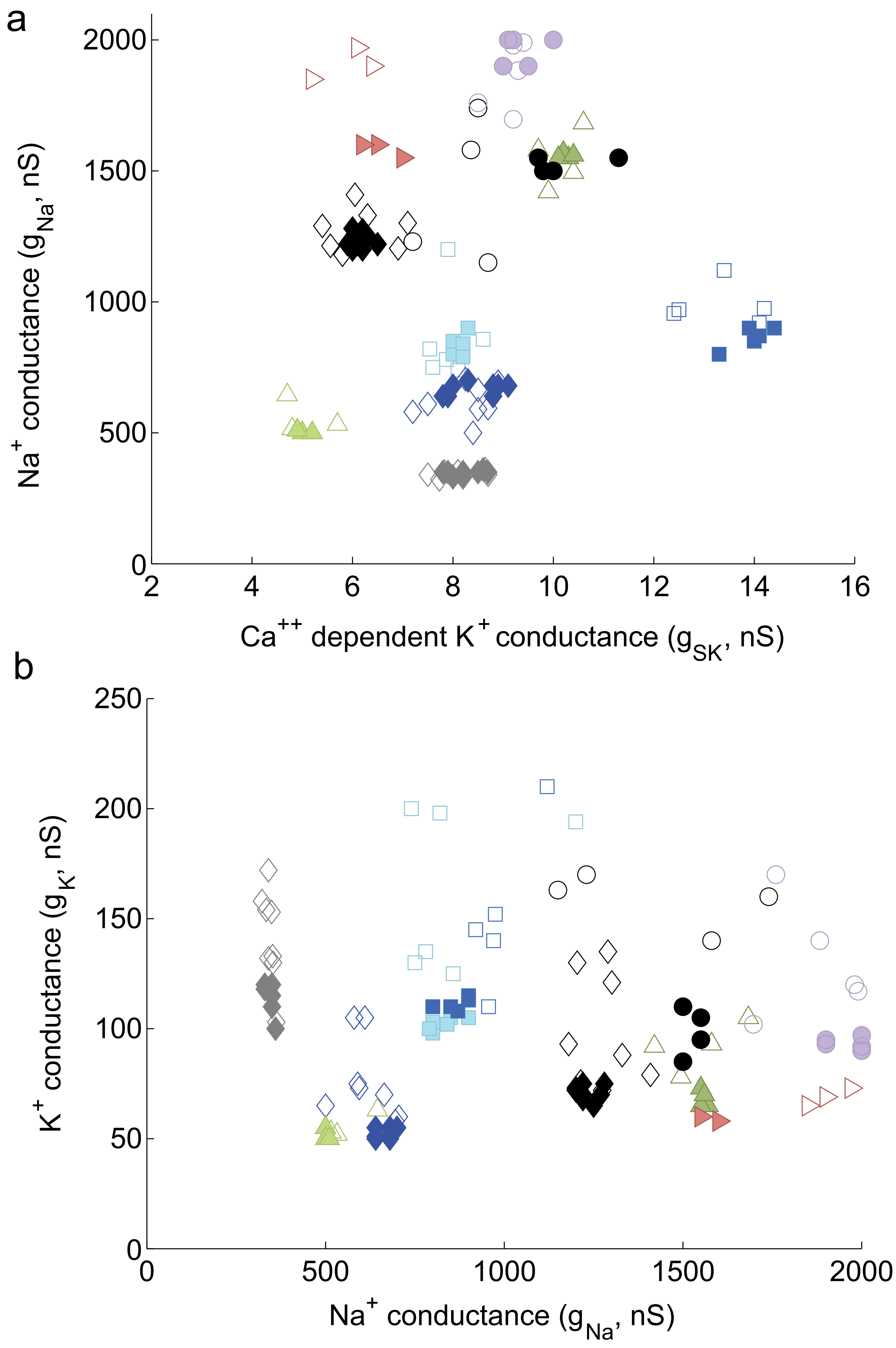

Supplementary Figure 7. Exhaustive parameter searches yield results similar to those of manual fitting. Scatter plots for 58 neurons: **a** g_{SK} versus g_{Na}, **b** g_K versus g_{Na}. Each color-symbol represents one bird, with the values determined by automated procedures represented by open symbols and the values determined by the manual fits represented by filled symbols. The maximal conductances corresponding to global minima generated by the automated procedures were similar for neurons of the same bird and different for different birds. The values from the two procedures are similar. The dispersion with each cluster (each bird) is greater for the automated than the manual procedures. The filled symbols correspond to those of Fig. 3c.

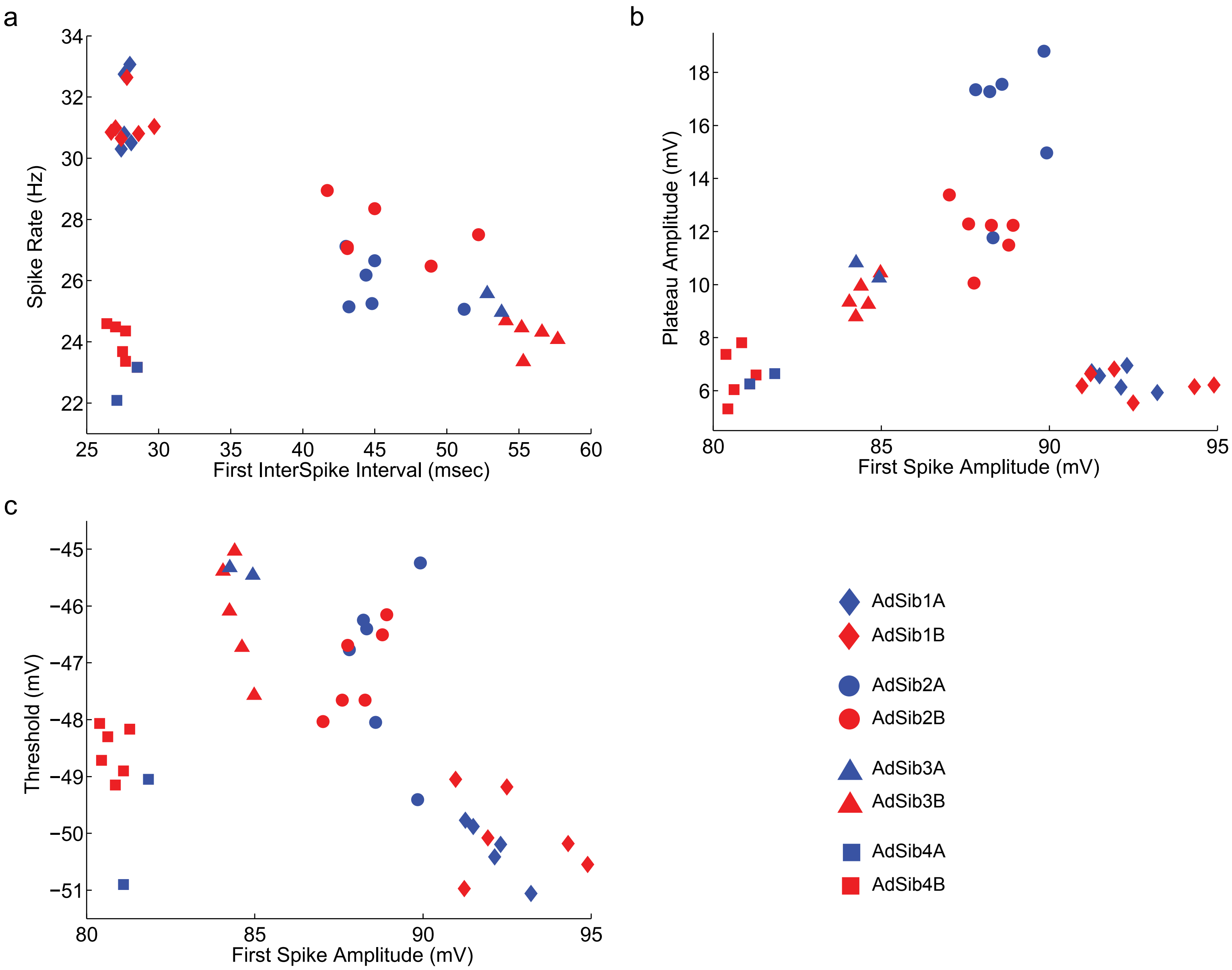

Supplementary Figure 8. Similar timing and morphological features of spike waveforms of neurons from adult sibling birds. a The timing features (first interspike interval, spike rate) of neurons show clear overlap among sibling pairs and distinct clusters across different pairs of siblings. Similar patterns of distribution are seen for the corresponding morphological features: **b** first spike amplitude versus plateau amplitude; **c** first spike amplitude versus threshold. Symbol-colors are the same as in Fig. 6c. Note the difference in scaling of the axes when comparing comparable data in Fig. 2c and Supplementary Fig. 3. That is, these four sibling pairs occupied a limited part of the total space occupied by all birds.

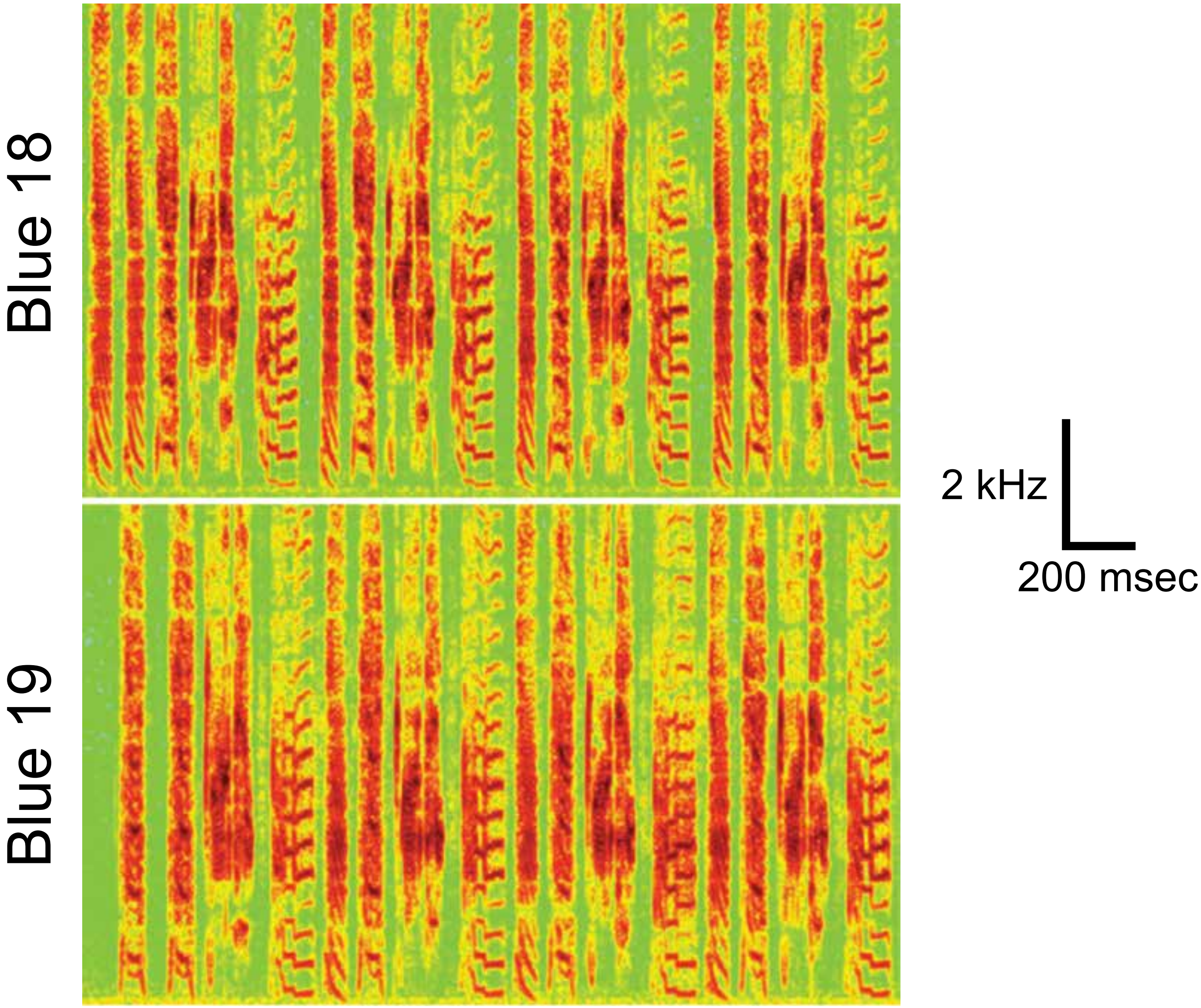

Supplementary Figure 9. Spectrograms of a pair of siblings showing the similarity in spectral and temporal features of their songs. Blue 18 (top) and Blue 19 (bottom) spectrograms show strong resemblance of spectral and temporal features of song. Note how similar is the timing of the syllables even over multiple motifs of singing. Note also that all four syllables were accurately copied. Blue 18 and Blue 19 are AdSib1a and AdSib1b, respectively, in Fig. 6.

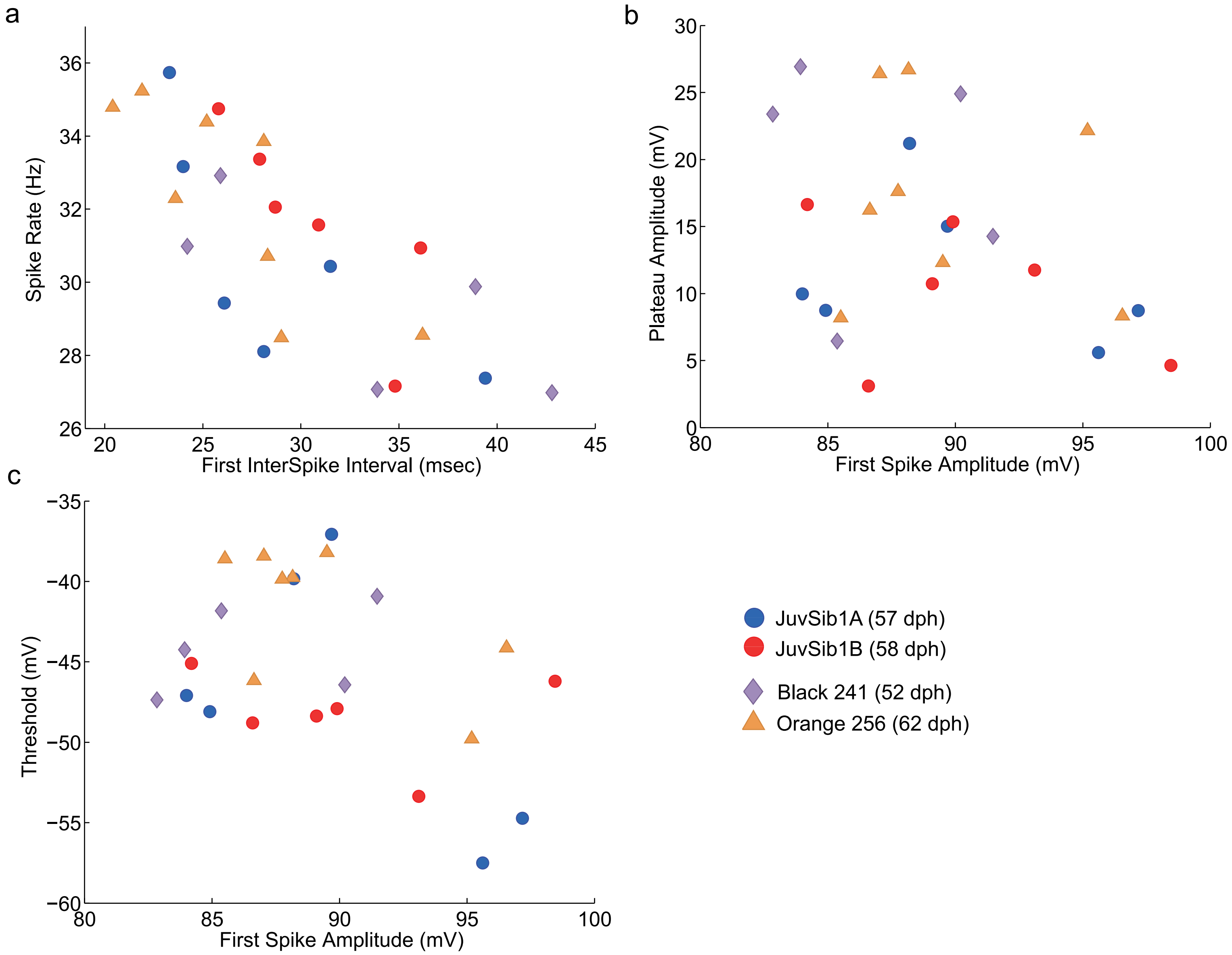

Supplementary Figure 10. Variable timing and morphological features of spike waveforms of neurons from juvenile birds. a The timing features (first interspike interval, spike rate) of neurons from juveniles show population trends, as expected, but note the lack of the distinct within-bird clustering pattern seen for adults. There is a similar lack of distinct within-bird clustering for juveniles when the corresponding morphological features are examined: **b** first spike amplitude versus plateau amplitude; **c** first spike amplitude versus threshold.
