# Supplementary material for: Intrinsic neuronal properties represent song and error in zebra finch vocal learning
Source: Nat Commun. 2020 Feb 19;11:952. doi: 10.1038/s41467-020-14738-7 (PMC7031510; doi:10.1038/s41467-020-14738-7)
Supplement: Supplementary file 3 — Reporting Summary [file 41467_2020_14738_MOESM3_ESM.pdf]

nature research

Corresponding author(s): Daniel Margoliash
Last updated by author(s): Dec 31, 2019

Reporting Summary

Nature Research wishes to improve the reproducibility of the work that we publish. This form provides structure for consistency and transparency in reporting. For further information on Nature Research policies, see Authors & Referees and the Editorial Policy Checklist .

Statistics

For all statistical analyses, confirm that the following items are present in the figure legend, table legend, main text, or Methods section.

n/a	Confirmed	
☐	☒	The exact sample size (n) for each experimental group/condition, given as a discrete number and unit of measurement
☐	☒	A statement on whether measurements were taken from distinct samples or whether the same sample was measured repeatedly
☐	☒	The statistical test(s) used AND whether they are one- or two-sided<br>*Only common tests should be described solely by name; describe more complex techniques in the Methods section.*
☐	☒	A description of all covariates tested
☐	☒	A description of any assumptions or corrections, such as tests of normality and adjustment for multiple comparisons
☐	☒	A full description of the statistical parameters including central tendency (e.g. means) or other basic estimates (e.g. regression coefficient) AND variation (e.g. standard deviation) or associated estimates of uncertainty (e.g. confidence intervals)
☐	☒	For null hypothesis testing, the test statistic (e.g. F, t, r) with confidence intervals, effect sizes, degrees of freedom and P value noted<br>*Give P values as exact values whenever suitable.*
☒	☐	For Bayesian analysis, information on the choice of priors and Markov chain Monte Carlo settings
☒	☐	For hierarchical and complex designs, identification of the appropriate level for tests and full reporting of outcomes
☒	☐	Estimates of effect sizes (e.g. Cohen's d, Pearson's r), indicating how they were calculated

Our web collection on statistics for biologists contains articles on many of the points above.

Software and code

Policy information about availability of computer code

Data collection	All source code that had been used in this work to analyze or produce figures will be freely available online on the corresponding author's lab website.
Data analysis	The computer code used to model the neurons in this study as well as the code for the brute-force fitting procedure will be available on the corresponding author's lab website.

For manuscripts utilizing custom algorithms or software that are central to the research but not yet described in published literature, software must be made available to editors/reviewers. We strongly encourage code deposition in a community repository (e.g. GitHub). See the Nature Research guidelines for submitting code & software for further information.

Data

Policy information about availability of data

All manuscripts must include a data availability statement. This statement should provide the following information, where applicable:

- Accession codes, unique identifiers, or web links for publicly available datasets
- A list of figures that have associated raw data
- A description of any restrictions on data availability

The datasets generated during and/or analysed during the current study are available from the corresponding author on reasonable request.

Field-specific reporting

Please select the one below that is the best fit for your research. If you are not sure, read the appropriate sections before making your selection.

☒ Life sciences ☐ Behavioural & social sciences ☐ Ecological, evolutionary & environmental sciences

Life sciences study design

All studies must disclose on these points even when the disclosure is negative.

Sample size	No sample-size estimation was performed beforehand. Once the structure of the novel effects began to emerge in the data, we limited recordings only to target HVCx and attempted to record as many HVCx in each animal as possible, in general achieving larger samples than have most other studies. The numbers of animals in each of the experimental groups (pharmacology, voltage clamp, juveniles/developmental, adult siblings) was limited by the labor intensive size of the overall project. We tried to record from at least four animals per group (or four pairs of sibs). We tried to record from as many animals as possible to examine the main effect (e.g. acoustics vs. IP).
Data exclusions	Experiments with unsuccessful surgery (failure of retrograde tracer procedures) or in rare cases when slice preparations were not viable (unhealthy slices) were excluded from the data. No recordings of HVCx were made in such cases, so there were no data to exclude.<br><br>Neurons with RMP > -65 mV or whose seal was lost during pharamacological manipulations were excluded from analysis (page 11, 1st paragraph).
Replication	*Describe the measures taken to verify the reproducibility of the experimental findings. If all attempts at replication were successful, confirm this OR if there are any findings that were not replicated or cannot be reproduced, note this and describe why.*
Randomization	Birds were randomly allocated into experimental groups.
Blinding	Blinding was applicable only in the sense of selecting the different birds randomly from the aviary within one of two age classes (adult, juveniles) or within the class of adult birds that had sibling males. Within each class no prerequisite knowledge of the bird's song was known or used to choose birds.

Reporting for specific materials, systems and methods

We require information from authors about some types of materials, experimental systems and methods used in many studies. Here, indicate whether each material, system or method listed is relevant to your study. If you are not sure if a list item applies to your research, read the appropriate section before selecting a response.

Materials & experimental systems

n/a	Involved in the study	
☒	☐	Antibodies
☒	☐	Eukaryotic cell lines
☒	☐	Palaeontology
☐	☒	Animals and other organisms
☒	☐	Human research participants
☒	☐	Clinical data

Methods

n/a	Involved in the study	
☒	☐	ChIP-seq
☒	☐	Flow cytometry
☒	☐	MRI-based neuroimaging

Animals and other organisms

Policy information about studies involving animals; ARRIVE guidelines recommended for reporting animal research

Laboratory animals	Juvenile male (15-90 d) and adult male (> 120 d) zebra fnches (Taeniopygia guttata) raised in the colony were used. Page 3, first paragraph; page 21, first paragraph.
Wild animals	NA
Field-collected samples	NA
Ethics oversight	All animal procedures were approved by the University of Chicago Institutional Animal Care and Use Committee (IACUC).

Note that full information on the approval of the study protocol must also be provided in the manuscript.
